# Damage-Detection Approach for Bridges with Multi-Vehicle Loads Using Convolutional Autoencoder

**DOI:** 10.3390/s22051839

**Published:** 2022-02-25

**Authors:** Kanghyeok Lee, Seunghoo Jeong, Sung-Han Sim, Do Hyoung Shin

**Affiliations:** 1 Research Institute of Construction & Environmental System, Inha University, Incheon 22212, Korea; kanghyeok@inha.ac.kr; 2 Advanced Railroad Civil Engineering Division, Korea Railroad Research Institute, Uiwang 16105, Korea; shjeong@krri.re.kr; 3 School of Civil, Architectural Engineering and Landscape Architecture, Sungkyunkwan University, Suwon 16419, Korea; ssim@skku.edu; 4 Department of Civil Engineering, Inha University, Incheon 22212, Korea

**Keywords:** damage detection, convolutional autoencoder, multi-vehicle loads, rigid-frame bridge, reinforced-concrete-slab bridge, deep learning

## Abstract

Deep learning has been widely employed in recent studies on bridge-damage detection to improve the performance of damage-detection methods. Unsupervised deep learning can be effectively utilized to increase the applicability of damage-detection approaches. Hence, the authors propose a convolutional-autoencoder (CAE)-based damage-detection approach, which is an unsupervised deep-learning network. However, the CAE-based damage-detection approach demonstrates only satisfactory accuracy for prestressed concrete bridges with a single-vehicle load. Therefore, this study was performed to verify whether the CAE-based damage-detection approach can be applied to bridges with multi-vehicle loads, which is a typical scenario. In this study, rigid-frame and reinforced-concrete-slab bridges were modeled and simulated to obtain the behavior data of bridges. A CAE-based damage-detection approach was tested on both bridges. For both bridges, the results demonstrated satisfactory damage-detection accuracy of over 90% and a false-negative rate of less than 1%. These results prove that the CAE-based approach can be successfully applied to various types of bridges with multi-vehicle loads.

## 1. Introduction

A structural-health-monitoring (SHM) system monitors the behavior of structures using multiple sensors, such as accelerometers and strain gauges on the structure [1]. With the SHM system, it is possible to measure behavioral changes in the structures caused by damage and to detect structural damage by analyzing these changes; this process is called damage detection. Damage-detection studies have been conducted for several decades to ensure bridge safety.

To detect damage with high accuracy, it is necessary to detect tiny changes in patterns created by each behavior before and after the damage. However, it is challenging identifying these changes based only on the bridge behavior obtained through monitoring. Therefore, damage-detection approaches utilize damage-sensitive features (DSFs) that are extracted by signal-processing techniques or statistical-analysis methods from the behavioral data obtained through raw monitoring and use them to identify changes in behavior patterns. Thus, a DSF, more sensitive to bridge damage than conventional ones, can potentially improve the performance of the damage-detection approaches.

Based on this potentiality, several damage-detection studies have been conducted to generate a DSF with excellent pattern recognition to detect damages through deep learning. Most studies utilize supervised deep learning to detect damage. In order to utilize supervised deep learning for damage detection, a large amount of bridge-behavior data obtained from various damage conditions of the bridge and label data is required, which indicate the damaged state of the bridge at the time the behavior data are obtained. However, obtaining such data from actual bridges is impractical, which limits the field applicability of damage-detection approaches based on supervised deep learning.

Unsupervised deep learning has the advantage of training the model using only the behavior data without any label data. Considering the merits of unsupervised deep learning, the authors proposed a damage-detection approach using a convolutional autoencoder (CAE), which is an unsupervised deep-learning network, based on previous studies [2,3]. In previous studies, the CAE losses from the behavior data of the undamaged prestressed-concrete (PSC) bridges were used as DSFs. It was theoretically confirmed that a CAE-based damage-detection approach using these DSFs can effectively detect bridge damage [2]. After the theoretical verification of the CAE-based damage-detection approach, it was applied to an actual bridge. Therefore, field experience has proven that the CAE-based approach can be used with high accuracy (over 95%) [3].

However, previously proposed approaches are limited, as they have been verified only for PSC bridges under a single-vehicle load. In other words, multi-vehicle-load cases are encountered more frequently in real bridges compared with single-vehicle-load cases. To improve the feasibility of the proposed approach, it should be improved to detect damage in bridges with multi-vehicle loads. Therefore, the development of an improved CAE-based damage-detection approach was prioritized in this study. In this regard, the architecture and hyperparameters of the CAE-based damage-detection models investigated in this study were redesigned.

In addition, since the results of previous studies were obtained only for PSC bridges, it is necessary to verify whether this CAE-based approach is applicable to other types of bridges as well. Therefore, the rigid-frame bridge and reinforced-concrete (RC)-slab bridge, which are the most commonly used bridge types in Korea [4], were selected for examining the wider applicability of the CAE-based damage-detection approach.

## 2. Related Studies

The DSFs are key factors in determining the damage-detection performance of a given approach. In the conventional bridge damage-detection approaches, referred to as model-based approaches, e.g., [5,6,7,8,9,10,11], modal properties representing the dynamic properties of structures are used as DSFs. However, the DSFs for modal-based approaches have the disadvantage of being sensitive not only to damage but also to changes in the external environment such as temperature. In other words, if the environmental conditions are not properly controlled at the time the behavioral data of the bridge are measured, then it would be difficult to infer the effect of damage solely from the DSFs. Whereas non-modal-based DSFs, which are mainly extracted based on statistical analysis or signal-processing methods, e.g., [12,13,14,15,16,17,18,19], are relatively less affected by environmental conditions than modal-based DSFs; thus, they can be more helpful in damage detection [3,20].

Meanwhile, the performance of a given damage-detection approach can be improved by using a DSF that is more sensitive to damage than an existing DSF. From this perspective, several recent studies have attempted to use deep learning for damage detection. According to the results of studies using deep learning [21,22,23], the features extracted through deep learning demonstrate better performance than the non-modal-based DSFs extracted from statistical analysis or signal processing.

In addition to the type of DSF, the methods used to analyze the differences in the DSFs obtained before and after the damage to bridge also influence the damage-detection performance. Analysis methods such as the Mahalanobis distance (MSD), e.g., [24,25,26,27], principal component analysis (PCA), e.g., [15,19,28], and artificial neural network (ANN), e.g., [29,30,31,32] are mainly used for damage-detection approaches. The MSD method has a limitation; as the dimension of the used data increases, the importance of each variable of the data cannot be taken into consideration. In contrast, methods based on PCA and ANN can detect damage by considering the importance of each data variable, using weight parameters. In particular, it was confirmed that using an ANN, which is the basis of deep learning, has the advantage of a more sophisticated and complex network than PCA.

Generally, a deep-learning architecture consists of feature extraction and classification networks. As an example of an approach using deep learning for the damage detection of bridges [23], a convolutional neural network was used as a feature-extraction network and an ANN as a classification network. In other words, damage-detection approaches based on deep learning can show better performance compared to MSD-based and PCA-based approaches when extracting the DSFs and using them to detect damage.

There are remarkable damage-detection approaches [21,22,23] using deep learning, and deep-learning models have been trained for damage detection using supervised learning. To train such a supervised deep-learning model for bridge-damage detection, a large amount of bridge-behavior data measured from various stages of the damage to bridge and the corresponding data (called label data) are required. Several field tests are required to obtain data from a real bridge, including damage to the intended bridge. However, considering the bridge’s safety and the cost of field tests, it is impossible to conduct them in practice. In other words, despite the advantages of the deep-learning approach, its application in damage-detection using supervised deep learning is limited for an existing bridge.

**Figure 1:**
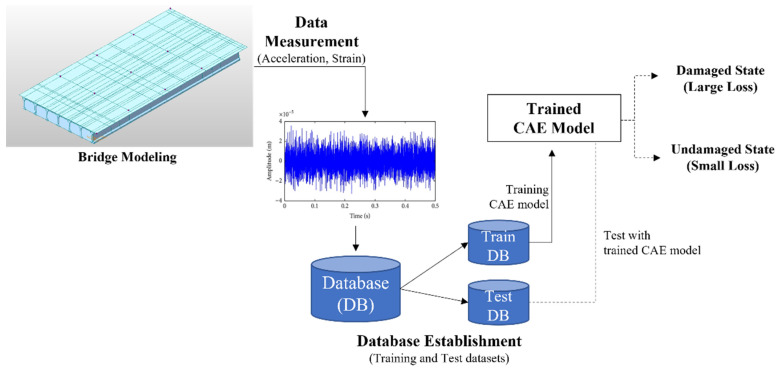
Data pipeline for CAE-based damage-detection framework.

To overcome this limitation, in this study, the authors proposed a CAE-based damage-detection approach based on previous studies [2]. First, a theoretical verification was conducted on a previous study. Figure 1 shows the verification process and data pipeline for the CAE-based damage-detection approach. A PSC bridge with the same size as the actual PSC-I bridge was modeled and simulated. Using this bridge model, acceleration data were obtained from a moving-vehicle load. Data obtained from the undamaged state of the bridge were used to train the CAE model for damage detection. The trained CAE model verified that the damage to the bridge with a single-vehicle load could be detected accurately. From the results, it was theoretically proven that the CAE-based approach can be applied to bridge-damage detection.

Because the CAE-based approach used in the previous study [2] was theoretically verified based on the simulation of a PSC-bridge model with a single-vehicle load, it was necessary to verify whether it could be applied in practice. Therefore, we conducted a field test of the CAE-based damage-detection approach that was proposed by the authors of a previous study [3] using actual data obtained from a PSC bridge.

Despite the satisfactory results obtained from both the theoretical verification and field experiments, the CAE-based approach presents a critical limitation, i.e., it can only be applied to bridges with a single-vehicle load. However, in practice, many situations exist in which multi-vehicle loads are used more frequently than single-vehicle loads in typical bridges. Hence, this study was performed to verify the performance of detecting damage in bridges with multi-vehicle loads in order to improve the applicability of the proposed approach. Studies pertaining to a specific type of bridge (PSC-I-type girder bridge) have been conducted. The applicability of the CAE-based damage-detection approach to other types of bridges should be verified. Therefore, the possibility of detecting damage to rigid-frame and RC-slab bridges using the proposed approach was investigated in this study.

## 3. Rigid-Frame Bridge

This study is based on the process proposed in a previous study conducted by the authors [2] and is designed to improve the CAE-based approach and to solve the problems highlighted in [2]. In the case of the approach proposed in [2], the accuracy of damage detection for the multi-vehicle-load situation was relatively poor compared to the single-vehicle-load situation. In addition, we studied whether the CAE-based approach operated properly even when the size of the bridge or the direction of the load changed.

### 3.1. Simulation Modeling

**Figure 2:**
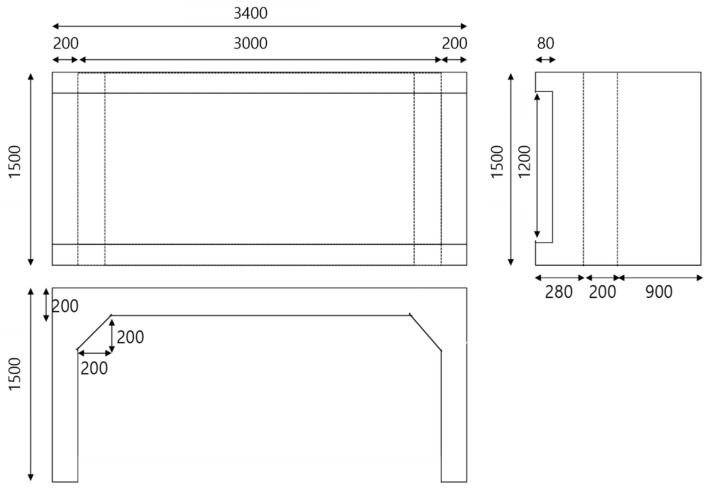
Dimensions of rigid-frame-bridge modeling (units: mm).

A rigid-frame bridge with a thickness of 200 mm, height of 1300 mm, deck width of 1500 mm, and deck length of 3400 mm, as shown in Figure 2, was modeled using the Midas Civil software, which is a commercial structural-analysis software. In this study, a linear, elastic finite-element model was considered for designing the target bridges. The model was designed to have a relatively smaller size than the RC-slab-bridge model to determine whether the CAE-based approach proposed in [2] could be utilized even if the size of the bridge was changed.

**Figure 3:**
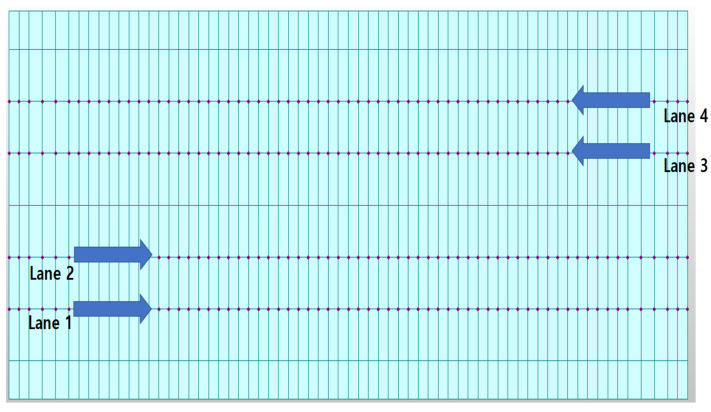
Traffic load points for rigid-frame-bridge modeling (Four lanes indicate the locations for the moving point load used to simulate the rigid-frame bridge.).

As shown in Figure 3, the positions and directions of the vehicle load on the bridge were designed to simulate the rigid-frame bridge and obtain its behavior data. In the case of Rahman-bridge model, there was a difference in size as compared to the absolute size of the bridge because the model was designed to have a smaller size than that of common bridges. Therefore, the movement speed of the point load was adjusted based on the size of the bridge model. The movement speeds of the point load were set to 21 different speeds (3.0, 3.1, 3.2, 3.3, 3.4, 3.5, 3.6, 3.7, 3.8, 3.9, 4.0, 4.1, 4.2, 4.3, 4.4, 4.5, 4.6, 4.7, 4.8, 4.9, 5.0 km/h). At every speed through each lane, 84 simulations (4 lanes × 21 speeds) were performed on the rigid-frame-bridge model. The vehicle load was idealized as a concentrated load or a one-point load, and a point load of 2 kN was applied to the node above the lane.

**Figure 4:**
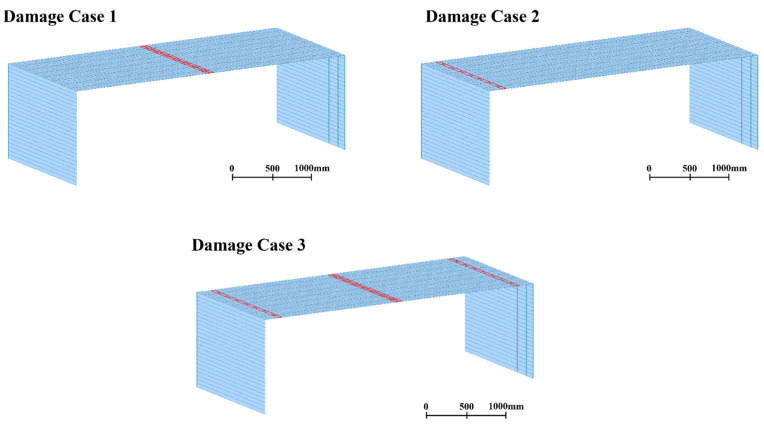
Damage locations of damage cases for rigid-frame-bridge modeling.

In the case of a rigid-frame bridge, damage occurs when the tensile forces occur, such as at the two ends and the middle of the bridge deck. In this study, the damage was simulated at these locations. As indicated by the elements in red on the bridge model in Figure 4, the damage locations were designated as damage cases 1–3, and the undamaged state of the bridge was designated as damage case 0. Damage case 1 was in the middle of the bridge deck, damage case 2 was on the left end of the bridge deck, and damage case 3 was at the two ends and middle of the bridge deck. In the process of modeling the bridge damage [33], the severities of damage cases 1–3 were set in such a way as to reduce the stiffness of the damage locations by 10% of the bridge stiffness of the undamaged state, which was 24.645 GPa. To simulate the target bridges in this study, a linear-time-history analysis was conducted to obtain the dynamic responses of the bridge model using the Runge–Kutta–Fehlberg method, which provides a numerical solution for ordinary differential equations.

**Figure 5:**
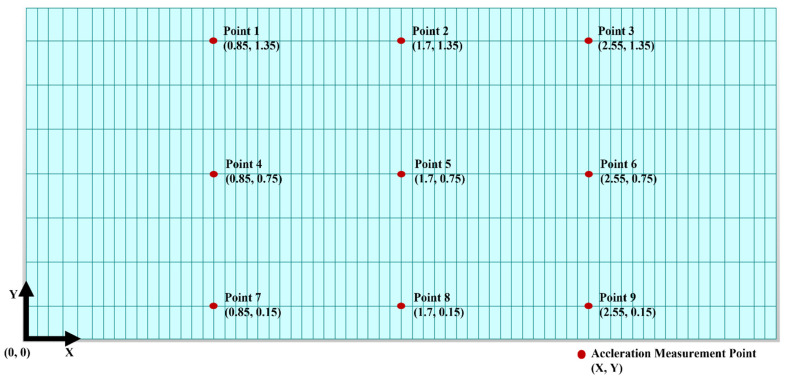
Acceleration measurement points on the bridge deck for rigid-frame-bridge modeling (Units: m).

The sensors were located at nine points on the bridge deck, as shown in Figure 5. The acceleration response of the bridge was obtained through a time-history analysis of the simulation of the bridge model. In this study, the acceleration response was called the base data. As a result of the simulations, 336 base-data points were obtained (four damage cases × 84 simulations).

### 3.2. Dataset

The rigid-frame-bridge model is a linear model; therefore, the acceleration response of the bridge shows a linear relationship with the vehicle load. Therefore, the multi-vehicle-load-case data for the rigid-frame bridge were created using a linear combination of the base data, according to the process proposed in [2].

Before performing the linear combination, the base data were preprocessed. The sampling rate of the 336 base data that were generated through simulation was 1000 Hz, and it was composed of time-series data with a length of 10 s considering the length of the bridge and the speed of the moving-vehicle load. The base data at 1000 Hz has too many data points per unit of time, which can reduce the training efficiency of the CAE model; the most time-consuming process in this study was the model training. To reduce the calculation cost of the CAE-model training, the sampling rate of the behavior data obtained from the bridge modeling was down-sampled from 1000 Hz to 100 Hz.

If down-sampling is performed at a low sampling rate, then the training efficiency of the CAE model increases. Considering the deep-network architecture, when the size of the input data is reduced by a factor of ten, the computational load is exponentially reduced. However, a large amount of information loss is inevitable. Such information loss may omit the behavioral characteristics of the structures contained in the data; thus, it is necessary to select an appropriate sampling rate. In this study, down-sampling was performed at an appropriate sampling rate in order to include up to the eighth mode of the rigid-frame bridge. Table 1 lists the natural frequencies of the first to eighth modes for all the damage cases. As shown in Table 1, the natural frequency of the eighth mode in the case of the undamaged bridge was approximately 44 Hz. The sampling rate of 88 Hz, which is the Nyquist rate or higher, should be considered. Therefore, down-sampling of the base data was performed using a sampling rate of 100 Hz.

Owing to the characteristics of the artificial neural network, it is easier to train an artificial neural network if the input data are normalized prior to being used [22]. The CAE is a methodology based on artificial neural networks, so good performance can be guaranteed by normalizing the input. Accordingly, in this study, 336 base data points were normalized. Max–min normalization was used as the normalization method.

The time-domain data of 5000 min for the multi-vehicle situation were generated by the linear-combination process. In this combination process, randomly chosen preprocessed base data for all lanes and vehicle speeds from each damage case were combined to generate the time-domain data for each damage case. The time-domain data for 5000-min, which was generated by linear combination for each damage case, were divided into 15,000 subsampled data points, and each subsampled data point had 2000 data points (20 s time-domain data) associated with it. Four datasets were generated and used to train the CAE model for damage detection, and each dataset included 15,000 subsampled data of each damage case.

Whereas data preprocessing was performed to increase the realism of each data point, random measurement errors that may appear during the actual measurements were intentionally applied. The applied measurement error was generated using two Gaussian distributions. The mean of the two distributions was zero, and the standard deviations were set to 5% and 10%. Therefore, in the case of no errors, three measurement errors were set and used for verification in this study.

In this study, only the dataset from the undamaged state of the bridge was used to train the CAE model for damage detection. The number of subsampled data points from the undamaged state of the bridge was 10,500, which is 70% of the total dataset. The remaining 30% of the 4500 subsampled data were used for testing the CAE model. However, the CAE model developed in this study can be considered as a classifier to distinguish two classes (damaged and undamaged). Unless the amount of data for each class used for verification is the same, it can cause a data imbalance and lower the reliability of the results. Therefore, even in the dataset of damage cases 1–3, 4500 subsampled data points from each dataset were randomly selected and used. This process was carried out in order to distinguish the undamaged state and each of the three damage cases (e.g., undamaged versus damage case 1; undamaged versus damage case 2; undamaged versus damage case 3), and all three measurement errors were applied. Therefore, nine sets of training and testing datasets were used.

### 3.3. CAE Model Architecture

**Figure 6:**
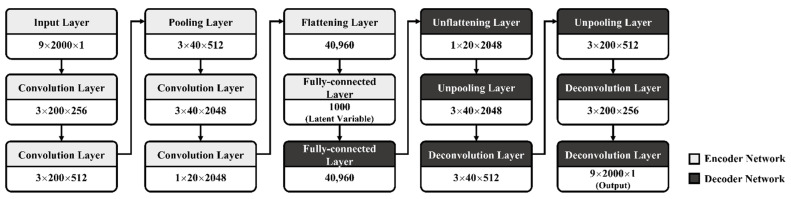
CAE architecture for rigid-frame-bridge-damage detection.

An improved CAE architecture for the development of CAE-based damage-detection models for bridges with multi-vehicle loads was designed, as shown in Figure 6. It was based on the architecture proposed by the authors in previous studies [2] and was also improved upon and used in this study. In general, CAE has two networks: an encoder and a decoder. The encoder of this CAE architecture had three convolution layers, two max-pooling layers and one fully connected layer. Conversely, the decoder of this CAE architecture had three deconvolution layers, two unpooling layers and one fully connected layer.

The size of the input data was designed to be 9 × 2000 (nine sensors × 20 s × 100 Hz), which is the matrix size of the subsampled data. In addition, the different input-data sizes and filter depths of the convolution layer were set to 256, 512, and 2048 for each of the three convolution layers. The size of the deconvolution filter was symmetrically applied to the convolution filter. In particular, the latent variable, which provides the most important contribution to output-data reconstruction, is a significant factor in determining the performance of CAE models. The reconstruction ability of the decoder in the CAE model directly affects the construction of the loss value, which was used as the input for damage detection. In a preliminary study, several nodes for latent variables were evaluated based on the CAE architecture shown in Figure 6, and the number of nodes for latent variables was empirically set to 1000 in this study, which is 1/18 of the input data size. Table 2 lists the hyperparameters of the CAE architecture that were configured for training the CAE-based damage-detection model.

The architecture and hyperparameters of the CAE models were designed and configured using Keras based on TensorFlow version 1.15 with CUDA version 10.2, which is a deep-learning framework, as well as Python programming. All training and test processes were performed in the following computing environment: Intel i7-8700K CPU, 32 GB DDR4 RAM, and two NVIDIA GTX-1080Ti GPUs. Software and hardware environments were applied to both bridge-type cases.

### 3.4. Results and Discussion

In this study, CAE model-training and testing were performed for nine combinations of the three damage cases and three levels of measurement errors (refer to Section 3.2). To evaluate the performance of the damage-detection model, the threshold for achieving the maximum accuracy for each combination of cases was determined using the trial-and-error method and the accuracy was calculated. From the results of the tests of the nine combinations, a maximum accuracy of 93% was demonstrated in the combination having both cases as damage case 3 and no measurement error, and the accuracies of the other combinations were over 91%. In other words, the CAE model for the rigid-frame bridge could be developed as a model that could achieve a maximum accuracy of over 91%. The maximum accuracy reflects the potential performance of the CAE-based damage-detection model; the maximum accuracies of the CAE-based models for the target bridge model were obtained preliminarily.

However, threshold calculation using this trial-and-error method cannot be performed in practice because information regarding the actual state of the bridge is not available. Therefore, a threshold that represents the accuracy closest to the maximum accuracy was determined in this study. Based on a preliminary study, five statistics (75th, 80th, 90th, 95th, and 99th percentiles), which are typically used as thresholds, were selected as candidates and then analyzed. Each threshold was obtained from the bridge-test dataset for each combination. The 80th percentile was selected based on the results of this study. Table 3 lists the accuracies and false-negative-rate (FNR) values for the nine combinations. The accuracies of all the combination cases were close to the maximum accuracy, and the FNR was within 1%. These results indicate that the bridge-damage-estimation model can be utilized effectively.

**Figure 7:**
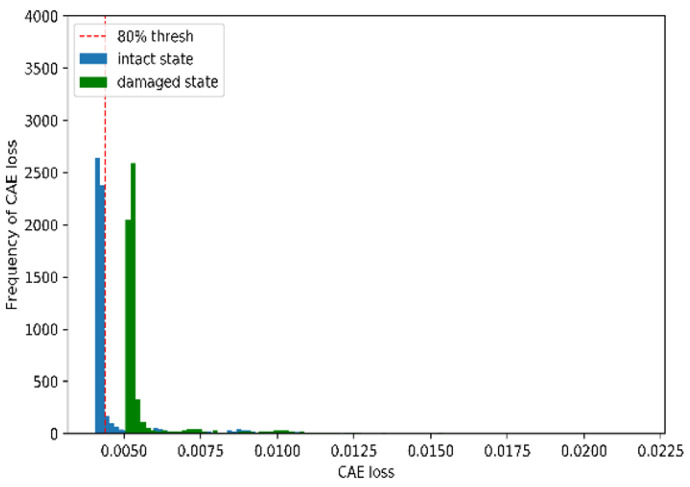
Example of histograms for CAE losses with each damage case and error level for rigid-frame-bridge modeling.

**Figure 8:**
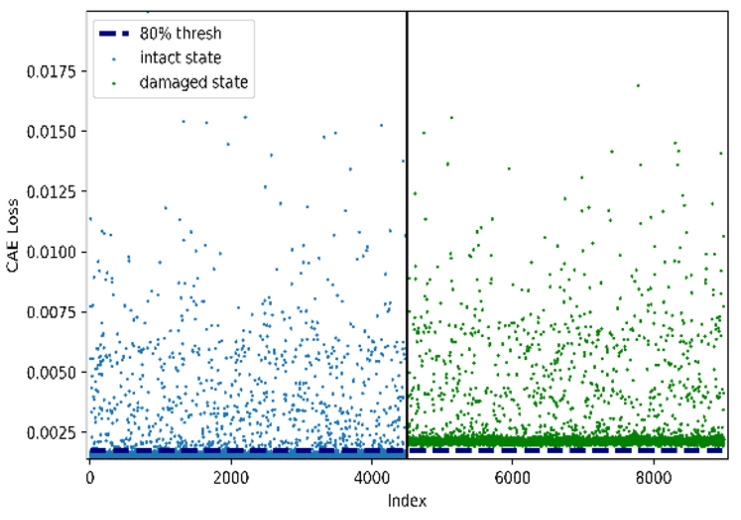
Example of scatter plots for CAE losses with each damage case and error level for rigid-frame-bridge modeling.

Figure 7 and Figure 8 illustrate the performance of the CAE-based damage-detection approach using the 80th percentile. Figure 7 and Figure 8 show examples of histograms and scatter plots of CAE losses obtained from both the undamaged and damaged states of the rigid-frame bridge. As shown in Figure 6 and Figure 7, the two distributions can be divided using the 80th-percentile threshold, which is represented by the dotted line in the figures. However, this indicates that both the distributions from the CAE losses of the undamaged-bridge data and the 80th-percentile threshold are close. Therefore, the threshold overlaps with the undamaged state, which means that the effect of increasing false positives (FPs) may occur, as well as a decrease in the damage-detection accuracy. Thus, it is necessary to consider this negative effect.

In general, the natural frequencies of the modes of a bridge were used to detect damage to the bridge as DSFs. The natural frequencies for damage detection were used as the input data for training an ANN classifier, and detection was performed with the trained classifier. As shown in Table 1, the difference in the natural frequencies for each mode is within 1%. However, it has been proved in the previous study [2] that it is difficult to accurately detect the damage to the bridge by the existing approaches of analyzing the natural frequency with the differences of 1% in the natural frequencies. From the additional verification of the ANN model that was trained with the same data as the CAE model, the accuracy was approximately 50%. This finding indicates difficulty in the damage-detection approach with the ANN, which is one of the most commonly used conventional approaches. Based on the results and these findings, the proposed CAE-based damage-detection approach can be used for the rigid-frame bridge with better accuracy than the existing approaches.

## 4. RC-Slab Bridge

The CAE-based approach applied to the rigid-frame bridge was verified with the case of an RC-slab bridge. In the case of the RC-slab bridge, the rigid-frame bridge’s architecture was utilized to verify whether the CAE architecture developed in this study can be used for other bridges.

### 4.1. Simulation Modeling

**Figure 9:**
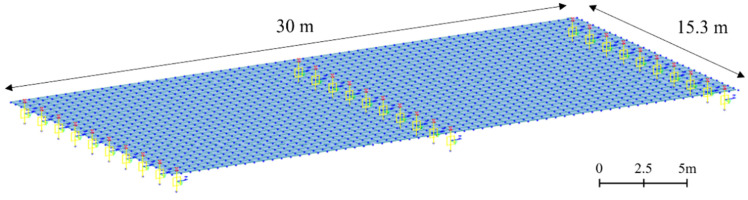
RC-slab-bridge modeling.

As shown in Figure 9, the size of the RC-slab-bridge model was designed to be 30 m × 15.3 m. Unlike the rigid-frame-bridge model, the RC-slab model was modeled as a two-span bridge. The Midas Civil software was used for RC-slab-bridge modeling, comprising a pier located in the central span, an abutment located at both ends, and a slab modeled with a plate element. For the boundary conditions, one side of the abutment was modeled with fixed support, and the rest with elastic-link support in order to closely replicate the actual bridge. In the case of bridge piers, the support comprised an elastic-link support.

**Figure 10:**
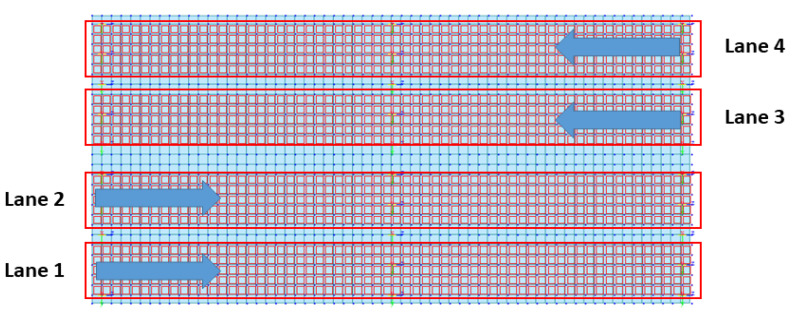
Directions of moving vehicles according to four lanes for RC-slab-bridge modeling (Four lanes indicate the locations for the moving point load (refer to Figure 11) used to simulate the RC-slab-bridge).

**Figure 11:**
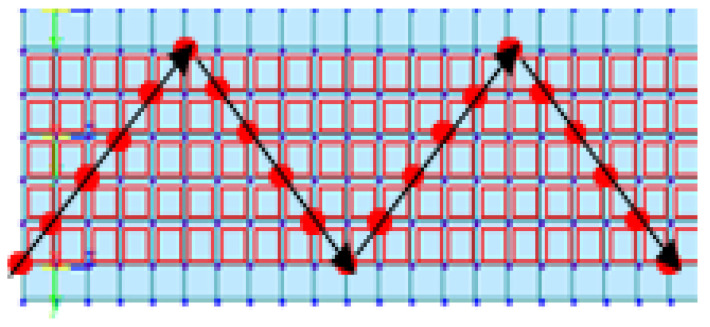
Example of movement of vehicle load for RC-slab-bridge modeling.

As in the case of the rigid-frame bridge, four lanes were designed to simulate the moving-vehicle load on the RC-slab-bridge model, as shown in Figure 10. The width of each lane was 3.6 m. In the test of the RC-slab bridge, we attempted to obtain bridge-behavior data by changing the direction of the vehicle load. Therefore, as shown in Figure 11, the point load of the moving vehicle was simulated to drive along a zigzag line. In addition, the point load simulating the vehicle was 1.5 tons. The movement speed of the point load was set to 15 different speeds (20, 25, 30, 35, 40, 45, 50, 55, 60, 65, 70, 75, 80, 85, and 90 km/h).

**Figure 12:**
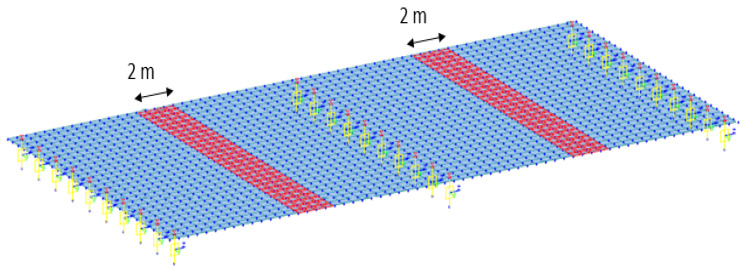
Locations of damage for RC-slab-bridge modeling.

As in the case of the rigid-frame bridge, the most vulnerable locations of the RC-slab bridge were chosen as the damage locations. The damage locations were set as the elements of the bridge that are in red in Figure 12 because they are the locations of the largest bending stress in the RC-slab bridge. The damage cases for the RC-slab bridge are listed in Table 4. The cases were divided according to the severity of damage. The damage severities were designed by reducing the elastic modulus of concrete in order to change the concrete stiffness of the red elements of the bridge model. The elastic modulus of the undamaged state of the bridge was 26.7 GPa. As summarized in Table 3, the damage severities were designed to be reduced by 3.3%, 6.9%, and 10.8% for damage cases 1, 2, and 3, respectively, compared with the elastic modulus of 26.7 GPa for the undamaged-concrete state.

### 4.2. Datasets

In the case of an in-service bridge, the occurrence of multiple-vehicle loads is more frequent than the single-vehicle loads. Therefore, in the case of the RC-slab bridge, as in the case of the rigid-frame bridge, we attempted to simulate a multiple-vehicle-load situation in which a large number of vehicles passed through a four-lane, two-way strip, which can improve the feasibility of the CAE-based damage-detection approach.

As in the case of the rigid-frame bridge, the base data were generated through a time-history analysis of the simulation of the bridge modeling. As a result of the simulation, 240 base data were obtained (4 damage cases × 60 simulations). According to Table 5, considering the Nyquist rate, the sampling rates should be greater than 50 Hz in order to include all of the characteristics from the first to the eighth mode. Therefore, the 240 base data were down-sampled to 100 Hz from 1000 Hz. The max–min method was used as the normalization method for the base data.

With the preprocessed base data, a linear combination was performed, and the time-domain data of 5000 min for the multi-vehicle situation in the RC-slab modeling were generated. Similar to the case of the rigid-frame bridge, the 5000 min time-domain data were divided into 15,000 subsampled data points with 2000 data points (20 s time-domain data). In addition, measurement errors were intentionally applied to the subsampled data in the form of randomly sampled errors that were extracted from two Gaussian distributions. This process was applied in accordance with each Gaussian distribution. The mean of the two distributions was zero, and the standard deviations were set to 5% and 10%. Therefore, the cases of no error and two different measurement errors were set and used for verification in this study.

A set of 10,500 subsampled data of the undamaged-bridge state was randomly selected and used for the training process of the CAE model. The remaining 4500 subsampled data points of the undamaged-bridge state and 4500 subsampled data points of the damaged-bridge state were used in the testing process of the CAE models. This was performed in order to distinguish the undamaged state from each of the three damage cases with the three measurement errors applied. Therefore, nine sets of training and testing datasets were used.

### 4.3. CAE Model Architecture

In practice, when detecting damage to a bridge, it is necessary to use a predefined model that can represent any bridge because the states of damage or no damage cannot be known. Therefore, in this study, a model with the same architecture as the CAE model applied to the rigid-frame bridge was used. The same hyperparameters and CAE architecture (see Figure 6) were used for the model training and validation of the RC-slab bridge, as described in Section 3.3. However, the CAE models for damage detection of the RC-slab bridge were trained using the RC-slab datasets. The computing environment for both training and testing was the same as that used in the case of the rigid-frame bridge.

### 4.4. Results and Discussion

The test for the RC-slab bridge was performed in the same manner as in the rigid-frame-bridge test (refer to Section 3.4). The maximum accuracies for the nine combinations of three damage cases and three levels of measurement error were verified. Consequently, it was confirmed that the maximum accuracy with the 95th-percentile threshold was 97.5%, and the maximum accuracy with the 99th-percentile threshold was approximately 99.5%. Combined with the case for the rigid-frame bridge, these results seem to be better because the RC-slab bridge has a relatively larger damaged-element area than that of the rigid-frame-bridge model. This results in the difference in behavior of the RC-slab-bridge modeling from the rigid-frame-bridge modeling, and the better performance of the RC-slab bridge.

However, if the CAE model is used for an in-service bridge, then it is important to determine and use a threshold that can guarantee the performance of the CAE-based damage-detection model. For the RC-slab bridge, five candidates for the threshold were verified in the same manner as in the case of the rigid-frame bridge. The 95th- and 99th-percentile thresholds showed the best performance. Table 6 lists the detection accuracies and FNRs when the 95th- and 99th-percentile thresholds were applied for the nine combination cases. When the 95th percentile was used as the threshold, all of the models showed highly reliable results with satisfactory damage-detection accuracies of 97.5% with 0% FNRs. In the case of the 99% percentile as the threshold, all but the combination of damage case 2 with the 10% measurement error showed accuracies of over 99% and 0% FNR. In the case of the combination of damage case 2 with the 10% measurement error, a satisfactory accuracy of 97.2% was obtained. These results indicate that the CAE model can be effectively used for the RC-slab bridge.

**Figure 13:**
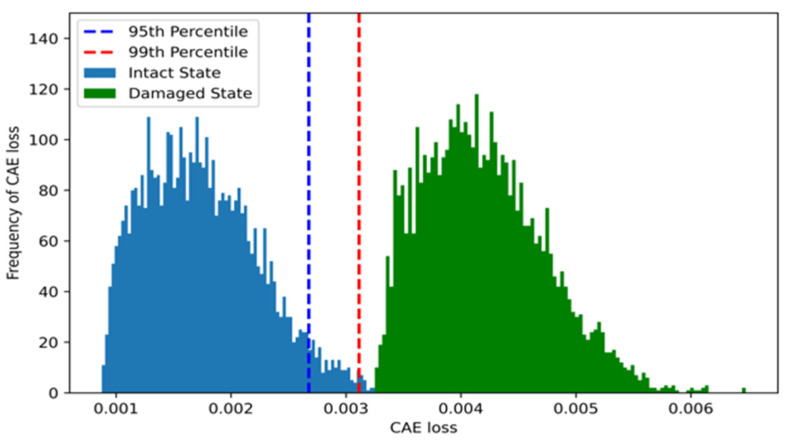
Example of histograms of CAE losses for RC-slab-bridge modeling.

**Figure 14:**
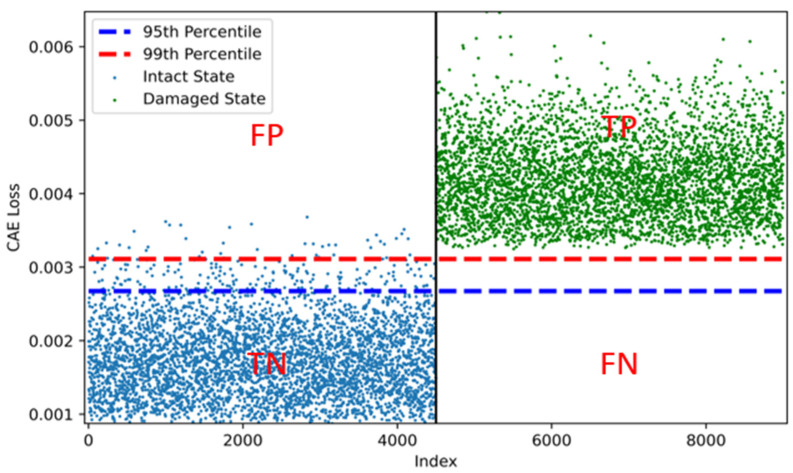
Example of scatter plot of CAE losses for RC-slab-bridge modeling (TP means true and positive. FP means false and positive. TN means true and negative. FN means false and negative. True and false indicates that the CAE model correctly and incorrectly predicted the bridge damage state, respectively. Positive and negative indicate the damaged state and the undamaged state, respectively, as the predicted state by the model.).

Figure 13 and Figure 14 show examples of a histogram and scatter plot illustrated by two distributions of CAE losses from both the undamaged and damaged states. Figure 13 and Figure 14 confirm the high accuracy obtained in this study. However, unlike the rigid-frame bridge, when the 99th-percentile threshold was used, the threshold was near the distribution that was extracted by the CAE losses of the damaged state. This means that there is a risk of increasing the FNR, which is the most important factor in the evaluation of damage detection, as well as a risk of decreasing the accuracy. In other words, in terms of the reliability of the CAE-based damage-detection model, we need to use only the 99th percentile as the threshold. Considering the difficulty in determining the threshold in practice, this could be a great way to use various threshold values for damage detection.

On the other hand, the mesh size of the RC-slab-bridge modeling was different from that of the rigid-frame-bridge modeling. Mesh size is one important factor in making a numerical model resemble an actual structure. If the research objective is to improve the accuracy, such as model updating, then the mesh size should be carefully considered. However, the numerical modeling in this study primarily focused on obtaining training and test datasets in order to verify whether the CAE-based damage-detection approach could be effectively utilized in the various damage scenarios in bridge modeling. Therefore, mesh-size selection was not a major research interest. The primary purpose of this study was to ascertain that CAE models could determine the different CAE losses according to each damage state of the bridge. Consequently, it was confirmed that the proposed approach could detect the damage with satisfactory accuracy, regardless of the numerical modeling of target bridges with different mesh sizes.

From the results and findings of the CAE-based damage-detection approach for both bridges, the possibility of detecting damages for the rigid-frame bridge and the RC-slab bridge, even though these bridges had different specifications and types, is high. Therefore, this indicates the potential of CAE-based damage detection to be used for various other types of bridges.

## 5. Conclusions

The aim of this study was to improve a CAE model that had been previously proposed [2] and to determine whether the CAE-based damage-detection approach is applicable to bridges with multi-vehicle loads, which comprise rigid frames and RC-slab bridges. For the development process of the CAE model, first the bridge was modeled and simulated. With bridge modeling, acceleration data were obtained from a moving-vehicle load. The acceleration data obtained from the undamaged state of the bridge were used to train the CAE model for damage detection. The trained CAE model verified that the damage to the bridge with multi-vehicle loads could be satisfactorily detected. The most time-consuming CAE-model training took approximately 2 d for each bridge model, and the simulation for obtaining the acceleration data took approximately 1 d for each bridge. The analysis of the results showed that the CAE-based damage-detection approach yielded good performances for both bridge types. In fact, it achieved satisfactory accuracies that exceeded 90% and 97% for the rigid-frame bridge and RC-slab bridge, respectively, with an FNR of approximately 0% for both cases. These results demonstrate the reliability and robustness of the improved damage-detection approach.

To improve the CAE model that was previously proposed, an effective method is to modify the number of nodes of the latent variable, which determines the reconstruction capability of the decoder in the CAE model. By improving the decoder capability, high accuracies of up to 27.1% were realizable, even when considering the relatively low damage severities in this study compared with the accuracies of the previous study. Although it may be premature to perform a direct comparison because of the different types of bridges, this improvement in accuracy demonstrates the potential of the improved CAE-based damage-detection model for bridges with multi-vehicle loads.

In addition, these findings indicate that the feasibility of the CAE-based damage-detection approach is satisfactory, regardless of the type, size, load direction, and number of bridge spans. In the specifications of both bridge models used in this study, the span length and width of the RC-slab bridge were 9 and 10 times larger than those of the rigid frame bridge, respectively. However, the CAE-based damage-detection models utilized the same architecture and hyperparameters for both bridge models and showed satisfactory accuracies that exceeded 90% and 97%, respectively. It may be premature to conduct an exact comparison owing to the different bridge types of both models; however, the proposed approach is expected to perform well with any bridge-modeling scale, considering the performance demonstrated based on the results of this study.

Meanwhile, it may be premature to assert whether the proposed approach is applicable to other bridges based on only two verification cases of bridges. However, because of the limitations of the simulation, such as time and cost, only two bridge cases were verified. Nevertheless, the proposed approach performed well for bridges of various types and sizes; consequently, it was confirmed that this approach is applicable to other bridges, provided that it affords high accuracy. In this study, multi-vehicle loads on bridges were investigated. Therefore, the proposed approach is expected to be feasible for various types of bridges.

However, it was difficult to select the appropriate threshold values in this study. Although the performance of the approach was satisfactory, to present a more reliable model, the CAE-based damage-detection approach must be improved. Therefore, the threshold value, which is the most important factor for calculating the accuracy, should be defined as a specific value; however, it was impossible to present the optimal threshold value based on the verification results for both bridges. Therefore, further experimental studies are necessitated to determine an optimal threshold for the CAE-based damage-detection approach. As a basis for future studies, this study may serve as a foundation as well as be utilized as an efficient method for bridge maintenance.

## Figures and Tables

**Table 1:** Natural frequencies of first- to eighth-order modes of rigid-frame-bridge modeling according to damage cases.

Mode Number	Undamaged Case Natural Frequency (Hz)	Damage 1 Case Natural Frequency (Hz)	Damage 2 Case Natural Frequency (Hz)	Damage 3 Case Natural Frequency (Hz)
1	7.316	7.316	7.313	7.311
2	9.128	9.109	9.124	9.101
3	18.146	18.139	18.128	18.103
4	21.157	21.137	21.149	21.121
5	24.312	24.309	24.309	24.302
6	32.372	32.359	32.366	32.346
7	40.813	40.744	40.774	40.668
8	44.593	44.489	44.586	44.476

**Table 2:** Hyperparameters of CAE-model training for the rigid-frame bridge and RC-slab bridge.

Batch Size	Regularization	Initialization	Gradient Method
256	L2 regularization	Xavier’s Initialization	RMSProp
Learning Rate	Learning Rate Decay Rate	Activation Function	Epoch
0.001	0.01	ReLU and tanh	Under 100

**Table 3:** Accuracies and FNRs of the CAE models corresponding to three levels of measurement errors and damage severity for rigid-frame-bridge modeling with the 80th threshold.

Damage Case	Measurement Error (Gaussian Distribution)		With 80th Percentile Threshold	
	Mean	Standard Deviation	Accuracy	FNR
1	No error		90%	0%
	0	5%	89%	1%
	0	10%	90%	0%
2	No error		89%	1%
	0	5%	89%	1%
	0	10%	89%	1%
3	No error		89%	1%
	0	5%	90%	0%
	0	10%	90%	0%
Average			89.4%	0.6%

**Table 4:** Elastic modulus and damage severity of damage cases for RC-slab-bridge modeling.

Damage Case	Elastic Modulus	Damage Severity
Undamaged Case	26.7 GPa	0%
Damaged Case 1	25.8 GPa	−3.3%
Damaged Case 2	24.8 GPa	−6.9%
Damaged Case 3	23.8 GPa	−10.8%

**Table 5:** Natural frequencies of modes for RC-slab-bridge modeling.

Mode Number	Intact Case	Damage Case 1	Damage Case 2	Damage Case 3
1	11.235	11.201	11.161	11.115
2	13.356	13.344	13.330	13.313
3	14.862	14.857	14.851	14.845
4	16.930	16.912	16.892	16.869
5	21.540	21.531	21.521	21.509
6	23.142	23.114	23.082	23.044
7	24.393	24.378	24.361	24.340
8	24.915	24.900	24.884	24.866

**Table 6:** Accuracies and FNRs of the CAE models corresponding to three levels of measurement errors and damage severity for RC-slab-bridge modeling.

Damage Case	Measurement Noise (Gaussian Distribution)		With 95% Percentile Threshold		With 99% Percentile Threshold	
	Mean	Standard Deviation	Accuracy	FNR	Accuracy	FNR
3	No error		97.5%	0%	99.5%	0%
	0	5%	97.5%	0%	99.5%	0%
	0	10%	97.5%	0%	99.5%	0%
2	No error		97.5%	0%	99.5%	0%
	0	5%	97.5%	0%	99.5%	0%
	0	10%	97.5%	0%	97.2%	0%
1	No error		97.5%	0%	99.5%	0%
	0	5%	97.5%	0%	99.5%	0%
	0	10%	97.5%	0%	99.5%	0%
Average			97.5%	0%	99.2%	0.3%
